# Impact of physical activity on energy balance, food intake and choice in normal weight and obese children in the setting of acute social stress: a randomized controlled trial

**DOI:** 10.1186/s12887-015-0326-7

**Published:** 2015-02-19

**Authors:** Antje Horsch, Marion Wobmann, Susi Kriemler, Simone Munsch, Sylvie Borloz, Alexandra Balz, Pedro Marques-Vidal, Ayala Borghini, Jardena J Puder

**Affiliations:** 1 Service Universitaire de Psychiatrie de l’Enfant et de l’Adolescent (SUPEA), Unité de recherche, Centre Hospitalier Universitaire Vaudois, 25 A, Rue du Bugnon, Lausanne, CH-1011 Switzerland; 2 Faculté des Sciences Sociales et Politiques, Institute des Sciences du Sport, Université de Lausanne, Lausanne, Switzerland; 3 Institute of Social and Preventative Medicine, University of Zürich, Zürich, Switzerland; 4 Department of Clinical Psychology, University of Fribourg, Fribourg, Switzerland; 5 Division d’Endocrinologie, Diabétologie et Obésité Pédiatrique, Département Médico-chirurgical de Pédiatrie, Centre Hospitalier Universitaire Vaudois, Lausanne, Switzerland; 6 Institut Universitaire de Médecine Sociale et Préventive, Université de Lausanne, Lausanne, Switzerland; 7 Service d’ Endocrinologie, Diabétologie et Métabolisme, Centre Hospitalier Universitaire Vaudois, Lausanne, Switzerland

**Keywords:** Childhood obesity, Physical activity, Energy balance, Food intake, Food choice, Stress, Parenting, Comfort food

## Abstract

**Background:**

Psychological stress negatively influences food intake and food choices, thereby contributing to the development of childhood obesity. Physical activity can also moderate eating behavior and influence calorie intake. However, it is unknown if acute physical activity influences food intake and overall energy balance after acute stress exposure in children. We therefore investigated the impact of acute physical activity on overall energy balance (food intake minus energy expenditure), food intake, and choice in the setting of acute social stress in normal weight (NW) and overweight/obese (OW/OB) children as well as the impact of psychological risk factors.

**Method:**

After receiving written consent from their parents, 26 NW (BMI < 90^th^ percentile) and 24 7-to 11-year-old OW (n = 5)/OB (n = 19, BMI ≥ 90^th^ percentile) children were randomly allocated using computer-generated numbers (1:1, after stratification for weight status) to acute moderate physical or to sedentary activity for 30 min. Afterwards, all children were exposed to an acute social stressor. Children and their parents completed self-report questionnaires. At the end of the stressor, children were allowed to eat freely from a range of 12 different foods (6 sweet/6 salty; each of low/high caloric density). Energy balance, food intake/choice and obesity-related psychological risk factors were assessed.

**Results:**

Lower overall energy balance (*p* = 0.019) and a decreased choice of low density salty foods (p < 0.001) in NW children compared with OW/OB children was found after acute moderate physical activity but not sedentary activity. Independent of their allocation, OW/OB children ate more high density salty foods (104 kcal (34 to 173), *p* = 0.004) following stress. They scored higher on impulsive behavior (*p* = 0.005), restrained eating (*p* < 0.001) and parental corporal punishment (*p* = 0.03), but these psychological factors were not related to stress-induced food intake/choice. Positive parenting tended to be related to lower intake of sweet high density food (−132 kcal, −277 to 2, *p* = 0.054).

**Conclusions:**

In the setting of stress, acute moderate physical activity can address energy balance in children, a benefit which is especially pronounced in the OW/OB. Positive parenting may act as a protective factor preventing stress-induced eating of comfort food.

**Trial registration:**

clinicaltrials.gov NCT01693926

The study was a pilot study of a project funded by the Swiss National Science Foundation (CRSII3_147673).

## Background

Behavioral, biological, social, and psychological risk factors play a role in the development of childhood overweight and obesity. Exposure to stress is an important risk factor [1-5]. Chronic hypersecretion of stress hormones can lead to an increase in body fat [6]. Mild to moderate acute stress exposure also influences food intake and choice in normal weight (NW) children [7]. These exposures, particularly to social stress (negative social interactions and interpersonal relationships that can aggravate social stigma and exclusion), occur frequently in children, and overweight children are especially targeted [8]. It is therefore important to find effective ways of coping with such stressors.

Physical activity represents a protective factor for the development of childhood obesity. It naturally increases energy expenditure [9] and can also regulate eating behavior via endocrine mediators such as insulin, leptin, and ghrelin [10-12]. Physical activity can influence total food intake in adults and adolescents [9,13], which further decreases overall energy balance. It also dampens the effect of stressors [14,15]. Reduced aversive affective states following physical activity might influence food intake or choice after stress exposure, for example by reducing the intake of comfort foods (high energy level sweet or salty foods). If there is a reduction or no compensatory increase in stress-induced food intake after physical activity, this could have a beneficial influence on overall energy balance in these stressful situations. Thus, physical activity is interesting for both obesity prevention and treatment.

So far, only one study in NW adults has focused on the impact of acute physical activity on food intake after acute stress exposure (computerized Stroop task) demonstrating that exercise before a highly demanding task reduced subsequent chocolate snacking in regular adult chocolate eaters [16]. Although this experiment successfully simulated work-related stress, it is less relevant for stressors encountered by overweight children who often experience stressful social situations that can augment stress-induced food intake. However, the impact of acute physical activity on food intake and choice after acute social stress has so far not been studied in a pediatric population.

Data from studies in older school children show that psychological risk factors, such as impulsivity, restricted eating behavior and specific parenting styles are related to increased body weight and childhood obesity [17-21]. There is a need to investigate the impact of these psychological factors on food intake and food choice under stress, in order to enhance obesity prevention and intervention efforts [22].

The role of impulsivity, conceptualized as a failure of attention, a failure to inhibit responses, and a failure to consider the probable negative long-term consequences of behavior (delay discounting or decision making), in influencing lifestyle choices has only recently become of interest [23,24]. Pre-pubertal obese children already display greater levels of impulsivity compared to healthy-weight peers, indicating that it may contribute to the onset and maintenance of obesity [25]. However, to what extent impulsivity is related to stress-induced eating in middle childhood has not been assessed so far. Food intake and choice after acute stress also depend on individual susceptibility to stress and/or eating behavior style [7,18]. For example, prepubertal NW and obese children with higher dietary restraint levels eat more after stress exposure and are more likely to choose high density sweet and fatty (comfort) foods [7,18,19]. There is also evidence that parenting styles can have an influence on childhood obesity; children whose parents have an authoritative parenting style as compared to another style eat more healthily, are more physically active, and have lower BMI levels [26,27]. However, research on the role of specific parenting techniques, such as positive parenting, monitoring, or corporal punishment in stress-induced food intake is lacking.

Given its potential importance for the prevention and treatment of childhood obesity, the current study investigated the impact of acute physical activity versus sedentary activity on overall energy balance, food intake and choice in response to acute social stress exposure in a sample of 26 consecutively recruited NW and 24 overweight/obese (OW/OB) children. Our primary hypothesis was that acute physical activity in children decreases energy balance in the setting of acute stress exposure. Pre-specified secondary hypotheses were: (a) Acute physical activity in children decreases the food intake and changes food choice in the setting of acute stress exposure; (b) Food intake and choice following acute stress exposure differ between OW/OB and NW children, independent of their allocation; (c) Eating behavior, impulsivity, and parenting style in OW/OB children are different from NW children and might impact food intake/choice in response to acute stress exposure.

## Methods

### Participant consent and recruitment

NW and OW/OB children were recruited from the general population through flyer advertisements and advertisements on the University Hospital Lausanne website. In addition, OW/OB children aged between 7 and 11 years were consecutively identified by clinicians working at the Children’s Hospital Lausanne and in the pediatric and emergency departments at the Hospital between September 2012 and March 2013; their parents agreed to be contacted by the research team. The research team subsequently phoned the parents of the identified children to verify their interest and to check that their children fulfilled the inclusion criteria: (a) BMI ≥ 90^th^ percentile for the OW/OB or BMI < 90^th^ for the NW according to WHO-criteria [27]; (b) aged between 7 and 11 years; (c) absence of chronic medical problems, such as epilepsy and asthma; (d) basic knowledge of French; and (e) physically able to perform a running exercise. After written consent on behalf of their children was obtained from the parents, an individual appointment was arranged. Participants received a gift voucher of CHF 50 to compensate for their time.

Of the 30 NW children who opted to participate, 4 did not participate in the experiment (due to illness or forgetting the appointment). Of the 52 OW/OB children contacted, 38 agreed to participate but 14 did not subsequently attend the appointment (due to illness or forgetting the appointment). Therefore, the final sample consisted of 24 OW/OB (5 OW and 19 OB) and 26 NW children. The clinical trial (clinicaltrials.gov NCT01693926) was approved by the ethics committee of the canton Vaud, Switzerland (protocol 286/2012).

### Study design and measures

#### Study design

**Figure 1:**
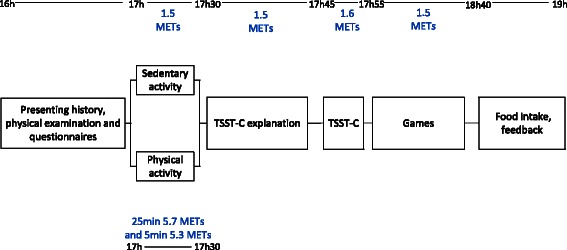
**Study design of the afternoon including the children-specific metabolic equivalents (METs) for all activities.**

Individual appointments took place between 4 pm and 7 pm at the University Hospital Lausanne (see Figure 1 for an overview of the study). Given the length of the experiment, parents were advised that their children should have a fruit snack at 3 pm. They subsequently did not eat anything until the end of the experiment when they could eat freely from a buffet (see below). On arrival, a physical exam was performed and parents then completed questionnaires in a separate room and left the children alone with the research team for the rest of the study. Children completed self-report questionnaires, when necessary with the help of the investigating psychologist. After stratification for weight status, they were randomly allocated (1:1, using computer-generated numbers by a staff member not involved in the activities or testing) to a moderate physical activity (n = 13 NW children and n = 12 OW/OB children) or a sedentary activity arm (n = 13 NW children and n = 12 OW/OB children) for 30 min and the allocation was concealed until the start of the activity.

In the acute *moderate physical activity arm,* children engaged in playful exercises with a basketball that included coordination, balance and speed with a physical education (PE) specialist. This was followed by a running exercise, involving a slalom using the ball and a basketball net, a short running competition against the PE specialist, and going up and down the stairs. Children’s heart rates were monitored throughout the intervention by a polar watch, aiming for a heart rate of 140 beats/min for the NW and 160 beats/min for the OW/OB children in order to correct for the increased oxygen consumption, i.e., higher exercise intensity in the OW/OB group at a given work output [28]. The PE specialist also ensured that the children’s perceived exertion was rated as “somewhat hard to hard” based on a repeated check every 5 min that rating on the categorized Borg scale was between 4 and 6 [29]. Children assigned to the *control arm* (sedentary activity) chose between playing calm board games, reading books, or drawing in the presence of the PE specialist. During the same time period, parents completed three questionnaires (see below).

Following this, the *Trier Social Test for Children* (TSST-C) was conducted for all children in both arms. The test has been developed to induce psychosocial stress and has been shown to elicit a strong and reliable stress response [30]. This standardized procedure consists of a 3-min preparation period, followed by a 5-min speech task and a 5-min mental arithmetic task adapted to the age and performance of the child. The speech and arithmetic tasks were filmed and performed in front of an audience of two experts.

At the end of the stressor, children were brought to the kitchen, where they were left alone for 20 minutes and were told that they could read, color in pictures, and/or eat freely from a range of 12 foods. They had comic books, games, and coloring material at their disposal. Parents and children were only aware that the study evaluated hunger after their activities, but not that food intake/choice were measured. At the end of the experiment, participants were debriefed.

#### Measures

##### Anthropometric measures

Body weight was measured in light clothes and without shoes to the nearest 0.1 kg with a digital medical scale. Standing height was assessed without shoes to the nearest 0.1 cm with a stadiometer. Children’s BMI was calculated and their attribution to the weight category confirmed (BMI ≥ 90^th^ percentile for the OW/OB or BMI < 90^th^ for the NW [27]). Waist circumference was measured by a flexible tape midway between the iliac crest and the lowest border of the rib cage.

*Energy balance* (kcal) was the primary outcome measure and was calculated for each child for the total duration of the experiment by subtracting energy expenditure from total food intake (see below).

##### Food intake and choice

Food was presented in 12 plastic cups, which were each filled to the top (Table 1). Salty and sweet food choices were provided in order to cover all food categories. Each type of food (sweet or salty) was further subdivided into high density (HD; high caloric content, “comfort food”) and low density (LD; low caloric content, “healthy food”) groups, leading to four categories. Thus, six cups contained salty foods, of which half contained HD (“salty (and fatty) comfort food”: salami, crisps (potato chips), peanuts) and half LD foods (bread, cherry tomatoes, rice cakes). The other six cups contained sweet foods, of which half were HD (“sweet (and fatty) comfort food”: milk chocolate, biscuits (cookies), gummy bears) and half LD (banana, apple, orange). The foods were weighed to the nearest 0.1 g before and after food intake to determine the amount consumed. Based on this information, the total number of calories (total food intake) and macronutrient (in grams) consumed was calculated for each child according to the Swiss Society of Nutrition guidelines [31].

**Table 1:** **Nutritional values for 12 food items offered**

Taste and density	Food items	Energy (kcal)	Proteins (g)	Lipids (g)	Carbo-hydrates (g)
**Salty**	Rice cakes	64	1.3	0.5	13.2
**LD**	White bread	132	4.7	0.6	26.1
	Cherry tomatoes	26	0.9	0.4	3.9
**Salty**	Crisps	145	1.3	10	12.5
**HD**	Peanuts	576	24.9	46.4	10.7
	Salami	194	12.7	15.9	0
**Sweet**	Apple	43	0.2	0.2	9.1
**LD**	Banana	72	0.8	0.2	15.9
	Orange	35.5	0.8	0.2	6.9
**Sweet**	Biscuits	188	2.6	8.3	25.3
**HD**	Milk chocolate	360	4.4	20.8	38.1
	Gummy bears	487	8.4	0	79

LD = low density; HD = high density.

Results are expressed on a per portion basis.

The mean total amount of calories presented was 2321 ± 39 kcal and the mean total amount of calories consumed 468 ± 246 kcal.

##### Energy expenditure

Energy expenditure (kcal) was calculated for each child for the total study period (4 hours) using child-specific metabolic equivalents (METs) taking into account the duration of the activities performed, as well as the children’s weight and age [32]. The children in the sedentary activity group spent 170 min in sitting activities corresponding to 1.5 METs and 10 min in a stressful activity (TSST-C, 1.6 METs). Children in the physical activity group spent 140 minutes in sitting activities (1.5 METs), 25 min playing games of at least moderate intensity in the form of basketball games and running exercises (5.7 METs), 5 min going up and down the stairs (5.3 METs) and 10 min in a stressful activity (1.6 METs).

### Children’s questionnaires assessing psychological risk factors

*Conners’ 3 ADHD Index- Child version* [33]: This questionnaire has 10 items measuring hyperactive/inattentive behaviors and those associated with mood/emotional lability. Each item is made up of behavioral description(s), which requires a rating on a 4-point Likert scale from 0 (‘not at all’) to 3 (‘very much’). Excellent psychometric properties have been reported [32].

Eating behavior was assessed using the *Dutch Eating Behavior Questionnaire* (DEBQ) [34]. This version contains 30 items measuring three types of eating behavior: ‘restrained eating’, ‘emotional eating’ and ‘external eating’. Children rated their responses on a 5-point Likert scale from 1 (‘never’) to 5 (‘very often’), which were summed up for each subscale. A stable factor structure, satisfying internal consistency and good test-retest reliability, has been reported and the DEBQ has been translated and validated for French Laguage use [35].

### Parents’ questionnaires assessing demographic information and psychological risk factors

Parents completed a brief demographic questionnaire including their place of birth, educational level, and actual work. Parental migrant status (assigned if at least one parent was born outside of Switzerland [36]) was assessed because migrant children are at a higher risk of obesity than their native counterparts [37]. Parental socio-economic status was calculated based on both parents’ educational history (1 = primary education, no professional training to 4 = university degree) and current profession (1 = unqualified employment to 4 = managing director or independent academic), with a maximum total score of 4 [38].

Children’s hyperactive/inattentive behaviors associated with deficient mood and/or impulse regulation capacities were assessed by the *Conners’ 3 ADHD Index–Parent version* [33]. This questionnaire consists of 10 items and has adequate psychometric properties [39]. The questionnaire is scored and used in the same way as the Conners’ 3 ADHD Index-Child version.

Parenting practices were assessed using the *Alabama Parenting Questionnaire* (APQ) [40]. This questionnaire contains 40 items and has seven subscales: ‘positive parenting’, ‘responsible parenting’, ‘authoritarian parenting’, ‘inconsistent parenting’, ‘parental involvement’, ‘corporal punishment’ and ‘poor monitoring/supervision’. Parents rated their responses on a 5-point Likert scale from 1 (‘never’) to 5 (‘almost always’). Adequate psychometric properties have been reported [29]. An official French version has been published (Essau et al., [40]).

### Data analysis

All analyses were performed using STATA version 12.0 (Stata Corp, College Station, TX, USA). Due to the lack of previous studies in this area, power analysis was based on a study that measured the 24-h energy intake of OB adolescents with and without undergoing acute physical activity [27]. Based on their data, we estimated to involve 25 children in each randomization arm in order to achieve a difference with a power of 80%. Differences between randomization arms (acute physical vs. sedentary activity) or weight categories (NW vs. OW/OB children) in baseline demographic and anthropometric characteristics were calculated using mixed linear or, in the case of parental migrant status, logistic regression models. Differences between randomization arms or weight categories in energy balance, food intake, and food choices in the setting of acute stress exposure were calculated using mixed linear regression models, adjusting for potential confounders related to childhood obesity, energy balance, or food intake, (such as age, gender, parental socio-economic status, migrant status) and for weight category or randomization arm, as applicable, as covariates. Differences between weight categories in impulsivity-hyperactivity, habitual eating behavior and parenting style were calculated using mixed linear regression models, adjusting for the same covariates including the randomization arm. In a last analysis, the adjusted impact of impulsivity-hyperactivity, habitual eating behavior, and parenting style on food intake and food choices in response to acute stress exposure was analyzed using mixed linear regression adjusting for the same covariates, including weight category and randomization arm. All differences are shown as beta-coefficients with 95% confidence intervals and data are presented as mean ± standard deviation, unless stated otherwise.

## Results

### Baseline sample characteristics

Table 2 shows the demographic data for the OW/OB and NW groups, randomly allocated to the acute physical or sedentary activity arm, thus forming four subgroups. All participants who entered the trial participated until the end. NW and OW/OB groups differed with regard to age (OW/OB children older), anthropometric measures (OW/OB children taller, higher waist circumference, higher weight and higher BMI), and parental SES (parents of OW/OB report lower SES), but there were no significant differences between the acute physical or sedentary activity arms.

**Table 2:** **Baseline demographic and anthropometric characteristics by randomization arm and weight category**

	Normal weight (n = 26)		Overweight/obese (n = 24)		Adjusted differences between	
Outcome variables	Sedentary (n = 13)	Physical activity (n = 13)	Sedentary (n = 12)	Physical activity (n = 12)	Randomization arms	Weight categories
Age (yrs)	8.5 ± 0.9	8.6 ± 0.6	9.6 ± 1.4	8.9 ± 1.2	−0.27 (−0.9 to 0.4)	0.7 (0.05 to 1.3)*
Girls/Boys (n)	7/6	6/7	6/6	9/3	1.4 (.5 to 4.2)	1.7 (0.5 to 5.2)
Height (cm)	134.4 ± 6.7	131.9 ± 5.6	143 ± 12	137.8 ± 11.4	−3.8 (−9.3 to 1.8)	7.3 (2 to 12.6)**
Waist circumference (cm)	57.5 ± 2.8	57.7 ± 11.5	82.2 ± 9.2	78.9 ± 9.6	−1.5 (−9.8 to 6.8)	23 (18 to 28)***
Weight (kg)	28.5 ± 3.9	28.4 ± 3.8	50.4 ± 9.2	43.6 ± 10	−3.3 (−10.1 to 3.5)	18.6 (14.3 to 22.8)***
BMI (kg/m^2^)	15.7 ± 1.1	16.2 ± 1.2	24.6 ± 3.3	22.7 ± 2.6	−0.63 (−3.2 to 1.9)	7.7 (6.4 to 9)***
BMI z-score	−0.15 ± 0.5	0.14 ± 0.7	3.13 ± 1.5	2.56 ± 1.1	−0.12 (−1.1 to 0.9)	2.85 (2.3 to 3.4)***
Parental SES	2.84 ± 0.6	3.11 ± 0.6	2.25 ± 0.8	2.5 ± 0.2	0.3 (−0.2 to 0.7)	−0.6 (−1 to −0.2)**
Parental migrant status yes/no (n)	5/8	7/6	3/9	2/10	0.8 (0.3 to 2.7)	3.3 (0.9 to 11.4)

*p < 0.05; **p < 0.01; ***p < 0.001.

BMI = body mass index; SES = socioeconomic status. The overweight/obese group includes 19 obese children.

Parental migrant status denotes children with at least one migrant parent. Results are shown as mean ± standard deviation, as odds ratios (95% confidence interval) for socioeconomic status or as differences (95% confidence interval) between randomization groups (children in the physical vs. the sedentary activity group) or weight categories (overweight/obese vs. normal weight children).

### Energy balance, food intake/choice in the setting of acute stress exposure

The mean total number of calories presented was 2321 ± 39 kcal and the mean total number of calories consumed 468 ± 246 kcal. After adjustment for confounders (see above), the overall energy balance during the afternoon was significantly lower in the children in the physical activity arm compared to the sedentary arm (*p* = 0.019, Table 3). Energy expenditure was obviously higher in the physical activity group. However, there was no compensatory increase in food intake in this group. Regarding food choice, children allocated to the acute physical activity arm were less likely to choose low density salty foods (*p* = 0.001) and had a tendency to consume fewer carbohydrates (*p* = 0.07).

**Table 3:** **Energy balance, food intake and food choices in the setting of acute social stress by randomization group and weight category**

	Normal weight (n = 26)		Overweight/obese (n = 24)		Adjusted differences between	
Outcome variables	Sedentary (n = 13)	Physical activity (n = 13)	Sedentary (n = 12)	Physical activity (n = 12)	Randomization groups	Weight categories
Overall energy balance (kcal)	314 ± 223	230 ± 178	363 ± 306	143 ± 247	−164 (−300 to −28)***	72 (−86 to 230)
Energy expenditure (kcal)	129 ± 18	187 ± 25	228 ± 42	287 ± 66	68 (50 to 86)***	81 (60 to 102)***
Food intake (kcal)	443 ± 228	416 ± 178	591 ± 330	430 ± 219	−96 (−232 to 40)	153 (−5 to 311)§
LD salty food (kcal)	57 ± 38	28 ± 17	53 ± 44	28 ± 31	−25 (−44 to −6)***	−4 (−26 to 18)
HD salty food (kcal)	133 ± 96	122 ± 77	205 ± 129	185 ± 124	−21 (−80 to 39)	104 (34 to 173)**
LD sweet food (kcal)	35 ± 26	46 ± 44	50 ± 40	36 ± 39	0.6 (−22 to 23)	−4 (−31 to 22)
HD sweet food (kcal)	218 ± 117	220 ± 154	282 ± 195	181 ± 149	−51 (−140 to 37)	57 (−46 to 160)
Protein (kcal)	11 ± 8	11 ± 5	16 ± 10	12 ± 7	−3 (−6 to 1)	5 (0.3 to 9)*
Lipids (kcal)	19 ± 12	18 ± 8	24 ± 12	22 ± 11	−2 (−8 to 4)	7 (0.3 to 14)*
Carbohydrates (kcal)	54 ± 26	50 ± 26	76 ± 46	46 ± 28	−17 (−36 to 2)	16 (−6 to 39)

*p < 0.05; **p < 0.01; ***p < 0.001, § *p* = 0.058.

LD = low density, HD = high density. *LD salty food* included bread, cherry tomatoes, rice cakes; *HD salty food* salami, crisps, peanuts; *LD sweet food* banana, apple and orange; *HD sweet food* milk chocolate, biscuits, gummy bears.

The differences (95% confidence intervals) between randomization groups (children in the physical vs. the sedentary activity) or weight categories (overweight/obese vs. normal weight children) are adjusted for age, gender, parental socioeconomic status and migrant status and for weight category and randomization group, respectively.

Independent of their allocation, OW/OB children had a tendency towards higher food intake following stress than NW children (*p* = 0.058). Overall energy balance did not differ according to weight status but OW/OB children had a higher energy expenditure (*p* = 0.001). OW/OB children were also more likely than NW children to choose high density salty foods (*p* = 0.004*),* and had a higher consumption of proteins (*p* = 0.037) and lipids (*p* = 0.042).

### Impulsivity-hyperactivity, habitual eating behavior, and parenting style and their impact on food intake/choice

After adjusting for potential confounders (see above), OW/OB children reported more impulsive behaviors (*p* = 0.005, Table 4) than NW children, but parent-reported impulsive behaviors were not different. OW/OB children scored significantly higher on restrained eating (*p* < 0.001), but there were no differences in the other eating behavior subscales. Most reported parenting styles did not differ between OW/OB and NW children except that the parenting style of parents of the OW/OB children included more corporal punishment (*p* = 0.03).

**Table 4:** **Impulsivity-hyperactivity, habitual eating behavior and parenting style by weight category**

	Normal weight (n = 26)		Overweight/obese (n = 24)		
Outcome variables	Sedentary (n = 13)	Physical activity (n = 13)	Sedentary (n = 12)	Physical activity (n = 12)	Adjusted differences between weight categories
**Children**					
*Conners’ 3 ADHD Index- Child version*	7.5 ± 2.8	7.5 ± 3.6	12.8 ± 5.3	10.2 ± 6.0	4.6 (1.5 to 7.7)**
*Dutch Eating Behavior Questionnaire*					
Restrained eating	9.9 ± 4.9	10.9 ± 6	17.3 ± 5.9	17.8 ± 8.7	8.4 (4 to 12.8)***
Emotional eating	7.8 ± 6.2	10 ± 5.8	5.9 ± 7.1	9.3 ± 9.4	1.1 (−3.2 to 5.3)
External eating	19.5 ± 7.3	20.5 ± 7	13.3 ± 8.4	16.8 ± 8	−2 (−6.8 to 2.9)
**Parents**					
*Conners’ 3 ADHD Index– Parent version*	11.6 ± 3.5	12.3 ± 4.4	10 ± 5.0	13.3 ± 6.5	1.2 (−2 to 4.4)
*Alabama Parenting Questionnaire*					
Positive parenting	4.5 ± 0.2	4.5 ± 0.4	4.4 ± 0.3	4.6 ± 0.4	−1 (−0.3 to 0.2)
Responsable parenting	3.9 ± 0.5	3.8 ± 0.3	3.8 ± 0.4	4.0 ± 0.5	-.2 (−1 to 0.5)
Authoritarian parenting	3.8 ± 0.4	3.7 ± 0.6	3.1 ± 0.8	3.9 ± 0.5	-.2 (−0.7 to 0.2)
Inconsistent parenting	2.5 ± 0.7	2.3 ± 0.6	2.6 ± 0.7	2.6 ± 0.5	-.3 (−0.2 to 0.7)
Parental involvement	4.2 ± 0.4	4.2 ± 0.4	4.2 ± 0.5	4.0 ± 0.5	-.04 (−0.4 to 0.3)
Corporal punishment	1.6 ± 0.5	1.6 ± 0.3	2.2 ± 0.7	2.1 ± 0.6	.4 (1 to 0.8)*
Poor monitoring	1.2 ± 0.2	1.3 ± 0.4	1.4 ± 0.4	1.3 ± 0.5	1 (−0.2 to 0.3)

*p < 0.05; **p < 0.01; ***p < 0.001.

TSST-C = Trier Social Stress Test for Children.

The differences (95% confidence intervals) between weight categories (overweight/obese vs. normal weight children) are adjusted for age, gender, parental socioeconomic status, migrant status and for randomization group.

No significant associations between the parents’ or children’s perception of impulsive behaviors or the three eating behavior subscales with stress-induced food intake/choice were found. Regarding parenting styles, positive parenting tended to be related to lower intake of sweet HD (comfort) food (−132 kcal, −277 to 2, *p* = 0.054) while no significant association was found between the other parenting styles and stress-induced food intake/choice.

After adjusting for multiple comparisons for the APQ and DEBQ subscales, the results related to the parenting styles were no longer significant (p ≥ 0.2) while the differences regarding eating behavior (restrained eating) remained unchanged (p < 0.001).

## Discussion

This randomized study found a significantly lower overall energy balance (*p* = 0.019) and a decreased choice of low density salty foods (p < 0.001) in 9- to 11- year old NW children compared with OW/OB children after acute moderate physical activity but not sedentary activity in the setting of acute social stress. In response to stress, OW/OB children ate more high density salty comfort foods, independent of their allocation to acute physical or sedentary activity. They scored higher on impulsive behavior, restrained eating and parental corporal punishment, but these psychological factors were not related to stress-induced food intake or choice. In contrast, positive parenting tended to be related to lower intake of sweet high density food.

To our knowledge, this is the first study investigating the effect of acute physical activity on energy balance, food intake or choice in NW and OW/OB children in the setting of acute stress. Physical activity significantly decreased the overall energy balance by 30% in NW and by even 60% in OW/OB children in the setting of an acute social stress. There was no compensatory increase in food intake in the physical activity group following stress exposure. In contrast, physical activity led to a non-significant decrease in food intake compared to the sedentary group. These findings provide some novel evidence that acute physical activity might have an adaptive role in modulating stress-induced food intake and energy balance. In the absence of stress, acute physical activity does not necessarily lead to an increase in food intake or appetite or appetite hormones despite an increase in energy expenditure; it can even reduce short-term food intake [41,42]. Some authors have called this observation a “decrease in relative energy intake (food intake minus energy expenditure)” and we confirm these findings in the setting of stress in NW and OW/OB children. So far, one study investigated the influence of acute physical activity on stress-induced chocolate intake in healthy NW adults who regularly eat chocolate and showed comparable results [15]. Our findings extend this data to children, to NW and OW/OB subjects and to a larger variety of food choices. However, we only demonstrated these effects within a short time span (one afternoon) and it is possible that they may be reversed beyond the hours immediately following the experimental manipulation.

Our data suggest that school-aged children who are generally exposed to frequent stressors, such as social/peer stressors, might benefit from engaging in targeted bouts of acute physical activity. For example, parents and other educators should encourage children to walk or bike to school, which might be especially important the morning of a presentation or an oral exam. In addition, school- or family-based prevention and treatment programs should include intervals of physical activity which can help children’s coping with stress, their stress-induced food intake, and overall energy balance.

Acute physical activity also changed food choice following acute social stress exposure. Although it is somewhat surprising that following acute physical activity both NW and OW/OB children were half as likely to choose LD salty foods, another study reported a relationship between higher psychobiological stress reactivity and higher intake of salty foods among women following the TSST [43]. Thus, the observed reduction in the intake of salty food in our study might be explained by a possible decrease in stress reactivity due to physical activity [44]. Although differences in subgroup analyses were not statistically significant (possibly due to the lack of power), we observed that acute physical activity reduced the intake of HD sweet comfort food in OW/OB by a mean of 101 kcal. However, one has to consider that children’s food preferences may have affected their choices beyond the physical attributes of the foods (i.e., sweet/salty, low/high caloric density); this was not assessed in our study.

The observed tendency for increased food intake following stress in OW/OB compared with NW children is in agreement with previous studies [10,12,25] and provides further evidence for the link between psychological stress and overweight [8]. The finding that OW/OB children were more likely to choose high density salty foods (comfort fatty foods) than NW children in a stressful context is novel. Previous research had demonstrated a higher consumption of high density sweet foods [25]. Our findings also demonstrate that fatty food by itself (without a sweet component) can act as a reward to compensate for stress in children. These results are in accordance with the comfort hypothesis, illustrating that following stress, OW/OB children make more unhealthy food choices, which could lead to or maintain obesity [25]. Therefore, future research should explore whether physical activity preceding a stressful event may play a helpful role in OW/OB children.

Regarding psychological risk factors, OW/OB children had significantly higher scores on impulsivity, restrained eating behavior, and parental corporal punishment than NW children, which is in line with previous studies of older children [6-8,17-21,25,26] and confirms the association between those factors and childhood obesity. The scores of self-reported impulsivity in NW children were comparable to those of children without ADHD and the scores in OW/OB children to those reported by children with ADHD [33].

However, these psychological risk factors were not related to stress-induced food intake or choice. Only one parenting technique, i.e., positive parenting, tended to be related to lower intake of sweet HD (comfort) food, independent of weight status or randomization. Positive parenting is characterized by warmth, fostering the development of self-regulation including clear rules and consequences. This is intuitively linked with self-regulation of food intake and there is a lower risk of responsiveness to external food cues, e.g., the sight and smell of food. Whether impulsivity is related to stress-induced eating has not been investigated in previous research, so our result of a lack of an association between impulsivity and stress-induced food intake/choice needs to be replicated by future studies, which should also incorporate more behaviorally based measures of impulsivity. The finding that restrained eating was not associated with stress-induced eating is in contrast to other studies [18,25,45,46]. However, previous research [18,45,46] involved older children or adults and offered only unhealthy (comfort) food options.

Parenting techniques have so far not been investigated in this setting. Our finding of an association between positive parenting and stress-induced food choice suggest that positive parenting can act as a protective factor preventing stress-induced eating of HD, more energetic comfort food and thus the development of childhood obesity. This has clear implications for parenting interventions for families of obese children [47].

This study has some limitations. Whether parents provided their children with a fruit snack prior to attending the appointment was not verified systematically. No manipulation check regarding the TSST-C was carried out in this study, although the standard and widely published protocol was adhered to. Whether children had entered the stage of puberty was not assessed. Staff who measured the food intake in order to calculate energy balance were not blinded to the participants’ allocation, which might have accentuatedthe group differences. However, those measurements were always done in the presence of another member of staff, thus reducing the risk of a bias. Power calculations showed that the sample size was sufficient to detect main effects, but not interactions. Nevertheless, our sample size is comparable to other studies of similar design including acute stress exposure in OW/OB children [18]. Most NW children were recruited by advertisements and most OW/OB in a clinical setting, which might have also had an influence on the results. It is possible that parents whose NW children were particularly interested in sports responded to the advertisement, thus attenuating the group differences. Moreover, the response rate in the OW/OB group was lower than in the NW group and a sampling bias can thus not be excluded. The level of regular exercise (as a potential confounder) was not measured. Furthermore, psychological risk factors were only measured by questionnaires, relying on self-report and parent-report. It would have been advisable to use the Conners’ teacher scale in addition to the Conners’ parent scale, but for logistic reasons, this was not possible. The strengths of the study are the stringent methodological design including a clearly defined physical activity intervention, stress test, and food exposure in both NW and OW/OB children, all of which are difficult to perform with prepubertal children.

The findings of this study have important clinical implications. They suggest that acute moderate physical activity may be a helpful intervention to address food intake after exposure to social stress or in anticipation of social stress, particularly in obese children. These children are likely to be frequently exposed to stressful situations and may therefore be more likely to choose unhealthy food to cope with stress. In addition, as we and others have shown, they are especially vulnerable to eat comfort food in response to stress. Future research should aim to replicate findings and to investigate the amount and frequency of acute physical activity needed in order to achieve a significant beneficial effect. It would also be important to test the effect of physical activity on eating behaviors in children during repeated acute stress situations.

## Conclusions

This randomized study showed for the first time that in a context of acute social stress exposure, moderate physical activity can decrease overall energy balance and unhealthy eating after stress in prepubertal children. OW/OB children are more at risk of eating HD comfort foods in response to stress, but they particularly benefit, twice as much as NW children, from the impact of moderate physical activity on energy balance. However, we only demonstrated these effects within a short time span (one afternoon) and it is possible that they may be reversed beyond the hours immediately following the experimental manipulation.
